# LncRNA PVT1 Is a Poor Prognosticator and Can Be Targeted by PVT1 Antisense Oligos in Gastric Adenocarcinoma

**DOI:** 10.3390/cancers12102995

**Published:** 2020-10-15

**Authors:** Yuan Li, Shumei Song, Melissa Pool Pizzi, Guangchun Han, Ailing W. Scott, Jiankang Jin, Yan Xu, Ying Wang, Longfei Huo, Lang Ma, Christopher Vellano, Xiaolin Luo, Robert MacLeod, Linghua Wang, Zhenning Wang, Jaffer A. Ajani

**Affiliations:** 1 Department of Gastrointestinal Medical Oncology, Unit 0426, The University of Texas MD Anderson Cancer Center, 1515 Holcombe Blvd., Houston, TX 77030, USA; 2016110187@stu.cmu.edu.cn (Y.L.); ssong@mdanderson.org (S.S.); MPPizzi@mdanderson.org (M.P.P.); awscott@mdanderson.org (A.W.S.); jjin@mdanderson.org (J.J.); xuyan000@hotmail.com (Y.X.); yingwang@mdanderson.org (Y.W.); lhuo@mdanderson.org (L.H.); LMa6@mdanderson.org (L.M.); 2 Department of Surgical Oncology and General Surgery, First Hospital of China Medical University, Shenyang 110001, China; 3 Department of Genomic Medicine, The University of Texas MD Anderson Cancer Center, Houston, TX 77030, USA; GHan1@mdanderson.org (G.H.); LWang22@mdanderson.org (L.W.); 4 Center for Co-Clinical Trials, The University of Texas MD Anderson Cancer Center, Houston, TX 77030, USA; CPVellano@mdanderson.org; 5 Ionis Pharmaceuticals, Inc., 2855 Gazelle Court, Carlsbad, CA 92010, USA; XLuo@ionisph.com (X.L.); RMacLeod@ionisph.com (R.M.)

**Keywords:** LncRNA, PVT1, antisense oligonucleotides, gastric adenocarcinoma and therapeutic target

## Abstract

**Simple Summary:**

LncRNA-PVT1 is upregulated in a variety of human cancers, to validate LncRNA-PVT1 expression in gastric adenocarcinoma (GAC), RNA-SCOPE of LncRNA-PVT1 was performed in a large cohort in GAC TMAs. LncRNA-PVT1 is upregulated in GAC tissues and correlated with larger tumor size, invasion depth and lymph node metastasis. In the peritoneal metastasis ascites cells of GAC, LncRNA-PVT1 level is also upregulated compared to normal adjacent tissues. LncRNA-PVT1 is a poor prognosticator as well as therapeutic target in GAC. Targeting PVT1 using PVT1 ASOs suppressed tumor proliferation and invasion in both vitro and vivo, thus provide a novel therapeutic strategy for GAC.

**Abstract:**

Gastric adenocarcinoma (GAC) is inherently resistant or becomes resistant to therapy, leading to a poor prognosis. Mounting evidence suggests that lncRNAs can be used as predictive markers and therapeutic targets in the right context. In this study, we determined the role of lncRNA-PVT1 in GAC along with the value of inhibition of PVT1 using antisense oligos (ASOs). RNA scope in situ hybridization was used to analyze PVT1 expression in tumor tissue microarrays (TMAs) of GAC and paired normal tissues from 792 patients. Functional experiments, including colony formation and invasion assays, were performed to evaluate the effects of PVT1 ASO inhibition of PVT1 in vitro; patient-derived xenograft models were used to evaluate the anti-tumor effects of PVT1 ASOs in vivo. LncRNA-PVT1 was upregulated in GACs compared to the matched adjacent normal tissues in the TMA. LncRNA PVT1 expression was positively correlated with larger tumor size, deeper wall invasion, lymph node metastases, and short survival duration. Inhibition of PVT1 using PVT1 ASOs significantly suppressed tumor cell growth and invasion in vitro and in vivo. PVT1 expression was highly associated with poor prognosis in GAC patients and targeting PVT1 using PVT1 ASOs was effective at curtailing tumor cell growth in vitro and in vivo. Thus, PVT1 is a poor prognosticator as well as therapeutic target. Targeting PVT1 using PVT1 ASOs provides a novel therapeutic strategy for GAC.

## 1. Introduction

Gastric adenocarcinoma (GAC) is a common malignancy, with more than 1 million new cases and around 780,000 deaths per year [1]. Although screening strategies for early detection have been successful in Japan, more than half of patients with newly diagnosed GAC have advanced-stage disease; these patients usually have a poor prognosis [2]. GAC is inherently resistant to therapy, or develops resistance after initial tumor regression. The empirical treatments that are common in the clinic need to be replaced by scientifically rational approaches that are based on molecular underpinnings. Even though these therapeutic targets may only be present in a small fraction of patients, strategies based on targeting only a subset of patients with GAC having the biomarker will lead to sparing unnecessary therapy for a larger group of patients.

The extensive role of long-non-coding RNAs (lncRNAs) in gastrointestinal cancers have been a recent focus of research [3]. LncRNAs are non-protein-coding transcripts that are longer than 200 nucleotides and are widely distributed in the genome; more than 90% of the human genome is composed of non-coding RNAs [4]. Although previously discounted as the “dark matter” of the genome, lncRNAs are now recognized as part of the regulatory machinery. There is also evidence that some lncRNAs are dysregulated in cancer and play an important role in epigenetic regulation, gene transcription regulation, and other essential biological processes that are implicated in tumorigenesis and invasion.

LncRNA plasmacytoma variable translocation gene 1 (PVT1) was first discovered in mouse plasmacytoma in the mid-1980s [5,6]. It was named for its frequent involvement in the chromosomal translocation of mouse plasmacytoma [5]. Subsequent studies found that its locus is also a common variant of human Burkitt’s lymphoma [7]. More recently, it has been shown to be involved in the development of many tumor types [8,9,10]. Both PVT1 and the well-known protein-coding gene MYC are co-located on the 8q24 chromosomal region, known as the gene desert, which contains a large number of risk alleles that are implicated in cancer [11,12]. PVT1 produces a wide variety of spliced non-coding RNAs, as well as a cluster of six annotated microRNAs: miR-1204, miR-1205, miR-1206, miR-1207-5p, miR-1207-3p, and miR-1208 [13], which can promote tumor proliferation [14,15], and the resistance of chemotherapy [16].

Many studies have demonstrated that PVT1 is upregulated in a variety of human cancers, including GAC tissues and cell lines [17,18], non-small cell lung cancer [19], cervical cancer [20], and colorectal cancer [21], and serves as an onco-lncRNA in several tumor types [10]. Its aberrant expression can be a relatively independent regulatory factor for promoting tumorigenesis and MYC [22,23,24]. The upregulation of PVT1 suggests that it can be used for the early detection of cancer, the prognostication of drug resistance, or as a therapeutic target [25,26].

Advanced nucleic acid chemistry has enabled the development of purely synthetic “nucleic acid drugs” that may retain key structural features of RNA or DNA that have been modified. Sophisticated nucleic acid chemistry methods have allowed the development of clinically safe and efficient “antisense” drugs [27]. Antisense oligonucleotides (ASOs) seem to be an effective and feasible means of targeting genes to treat cancer. Several ASOs are being evaluated in clinical trials or have been approved by the FDA [28]. We recently reported that PVT1 ASOs can suppress esophageal cancer cell growth in vitro and in vivo [10]. However, it is unknown whether targeting PVT1 using PVT1 ASOs is efficient in GAC.

In the present study, we used RNA scope (R-Scope) in situ hybridization (ISH) and confirmed that PVT1 overexpression was significantly associated with larger tumor size, deeper tumor invasion, lymph node metastases, and a short survival duration in paired GAC and adjacent normal tissue samples from this large cohort of GAC patients (*n* = 792). Further, we demonstrated that by targeting PVT1, PVT1 ASOs inhibited patient derived GAC cell growth and invasion in vitro and in vivo. Thus, the results of our study suggest that lncRNA PVT1 is a prognosticator of short survival and could be a therapeutic target to pursue.

## 2. Results

### 2.1. Genomic and Transcriptomic Alterations of lncRNA PVT1 in GAC Tissues

**Figure 1:**
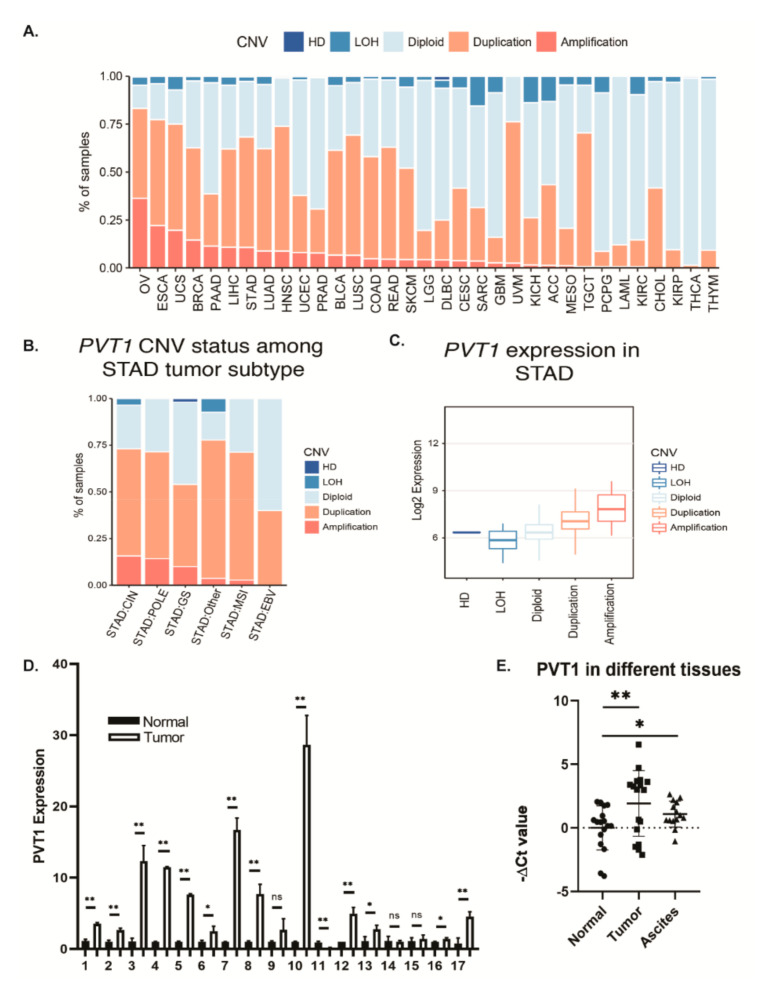
LncRNA PVT1 is amplified and overexpressed in GAC tissues compared to in normal tissues. (**A**) Analysis of PVT1 genomic alteration in the TCGA dataset revealed that PVT1 was amplified in over 15% of STAD (GAC) cases; around 70% of STAD cases contained both amplification and duplication. (**B**) Amplification of PVT1 gene in the CIN subtype was more common than in other subtypes. (**C**) PVT1 expression levels were significantly higher in amplification and duplication cases than in LOH and diploid cases. (**D**) PVT1 lncRNA mRNA was measured by qPCR and normalized to GAPDH in 17 pairs of primary tumor tissues and adjacent normal tissues. (**E**) qPCR of normal tissues, primary tissues, and ascites cells showed that the PVT1 mRNA level was upregulated in tumors and peritoneal metastases. * *p* < 0.5; ** *p* < 0.01; ns: not significant.

We first evaluated the genomic alteration of PVT1 in GAC tissues from TCGA dataset across different tumor types. Stomach adenocarcinoma (STAD) (same as GAC) had one of the highest PVT1 alterations among various tumor types, with (about 15% PVT1 amplification); ~70% of STAD cases contained both amplification and duplication (Figure 1A). We further evaluated PVT1 alterations (amplification and duplications) in different STAD subtypes such as genomic stable (GS), microsatellite instability (MSI), chromosomal instability (CIN), DNA polymerase epsilon (POLE), Epstein–Barr virus (EBV), and others. We found that CIN patients had a higher frequency of PVT1 amplification (Figure 1B). When integrated with the RNA sequence data for STAD, duplication or amplification of PVT1 was significantly associated with increased PVT1 expression (Figure 1C) indicating that genetic alterations of PVT1 in GAC led to upregulation of PVT1 expression in GAC tissues.

To further confirm TCGA results, we performed qPCR to measure PVT1 expression in 17 pairs of GAC tumor and normal tissues. PVT1 expression was highly upregulated in 15 out of 17 GAC tissues compared to normal adjacent tissues (Figure 1D). We also evaluated ascites cells from 14 PC cases from GAC patients and found that the PVT1 level was upregulated in these PC cells (*p* = 0.0497) as well (Figure 1E).

### 2.2. Upregulation of PVT1 lncRNA in GAC Tissue Was Associated with Poor Prognosis

**Figure 2:**
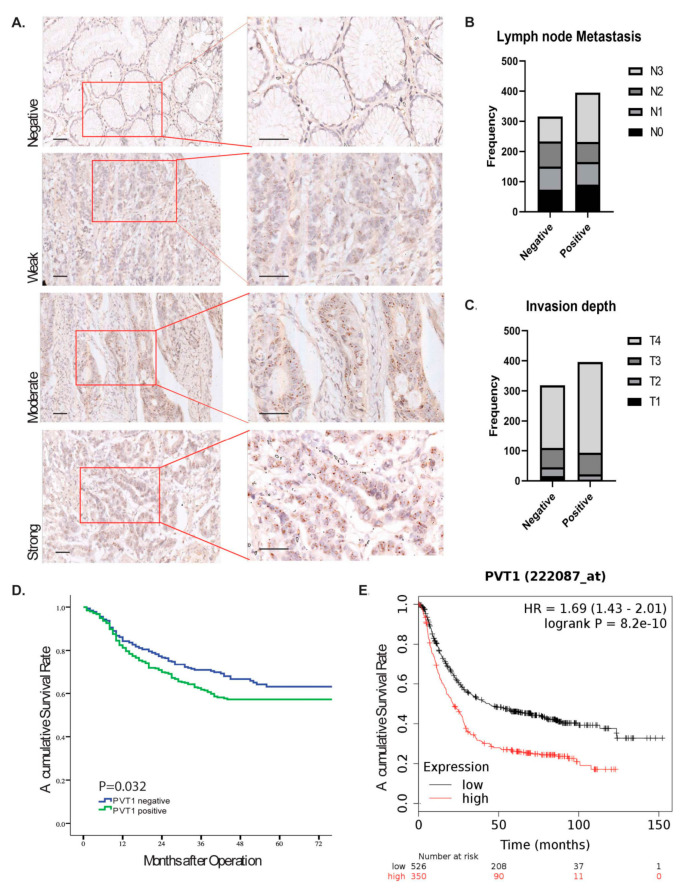
Positive expression of PVT1 in TMAs. (**A**) R-Scope ISH was performed using the PVT1 probe in a GAC TMA that consisted of 792 pairs of primary tumor tissues and adjacent normal tissues. Scale bars = 40 μm (**B**) PVT1 positivity in GAC tissues was correlated with lymph node metastasis. (**C**) PVT1 positivity in GAC tissues was correlated with advanced T stage. (**D**) Kaplan–Meier analysis of OS duration according to PVT1 expression in GC TMAs. GAC patients with positive PVT1 expression had shorter survival durations than did those with of low expression (*p* = 0.032). (**E**) Kaplan–Meier curve of the cohort from KMplot.com showed that positive PVT1 led to a shorter survival duration (*p* < 0.001).

To determine the relationship between PVT1 expression and patients’ clinic-pathologic features and outcomes, we performed R-Scope ISH on a large GAC TMA that contained 792 pairs of GAC and normal tissues. R-Scope ISH revealed that PVT1 was expressed primarily in cell nuclei (Figure 2A): the positive rate was 51.8% (410 of 792) in GAC tissues and 3.6% (28 of 787) in normal tissues.

PVT1 expression was significantly associated with clinical variables, as shown in Figure 2B,C and Table 1. High PVT1 expression was significantly associated with larger tumor size (*p* = 0.017), higher T stage (*p* < 0.001), and higher N stage (*p* = 0.008). The Kaplan–Meier curves revealed that patients with GAC who had high PVT1 expression had a shorter overall survival (OS) duration (*p* = 0.032) (Figure 2D and Table 2). We further validated this phenomenon in a separate patient cohort and found that PVT1 upregulation was associated with a shorter survival duration (*p* < 0.001) (Figure 2E; [29]). Univariate Cox regression analysis showed PVT1 expression is significantly associated with the high risk (HR1.312, with 95% CI:1.022–1.685, *p* < 0.05), however, the multivariate Cox regression analysis showed PVT1 is not an independent prognostic factor (Table 2).

### 2.3. Specific PVT1 ASOs Reduced PVT1 Expression in GAC Cell Lines

**Figure 3:**
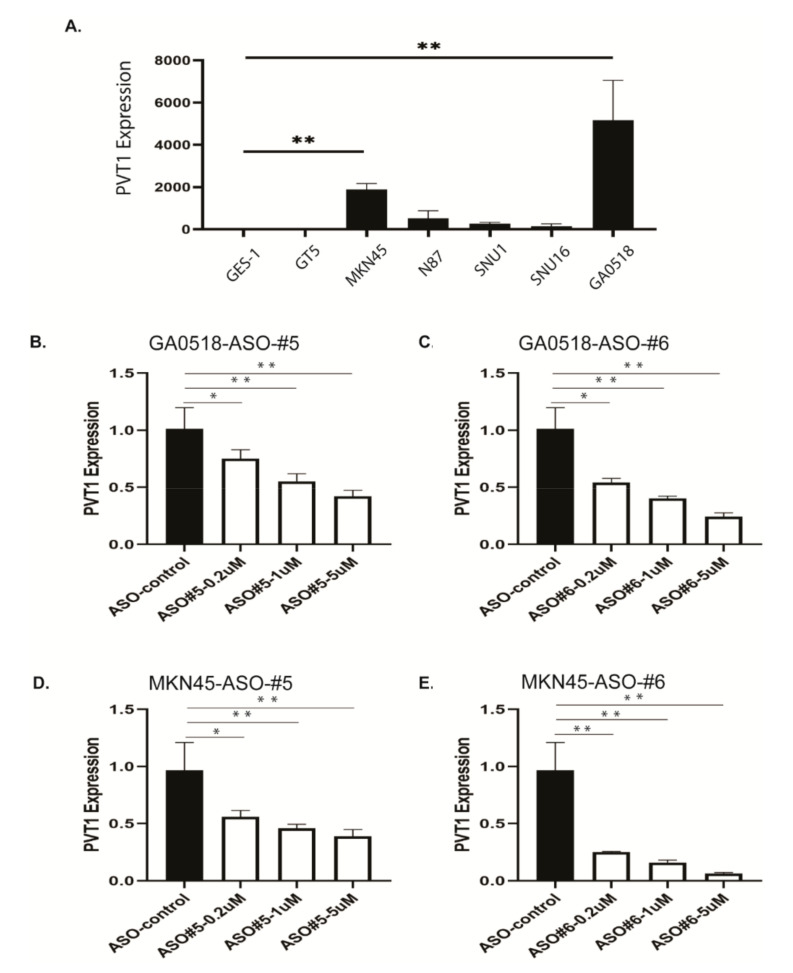
PVT1 expression level was reduced in ASO-treated GAC cells and ascites cells. (**A**) PVT1 expression levels in a normal cell line (GES-1), GAC cell lines (GT5, MKN45, N87, Snu-1, and Snu-16) and patient-derived ascites cells (GA0518) were determined by qPCR. (**B**,**C**) PVT1 expression levels were analyzed by qPCR in ASO-treated GA0518 cells. PVT1 expression levels were reduced in a dose-dependent manner by ASO#5(*p*_ctrl vs.0.2_ = 0.047) (**B**) and ASO#6(*p*_ctrl vs.0.2_ = 0.0124) (**C**), (**D**,**E**) PVT1 expression levels were analyzed by qPCR in ASO-treated MKN45 cells. PVT1 expression levels were reduced in a dose-dependent manner by ASO#5(*p*_ctrl vs.0.2_ = 0.046) (**D**) and ASO#6(*p*_ctrl vs.0.2_ = 0.007) (**E**) * *p* < 0.5; ** *p* < 0.01.

Expression of PVT1 was first detected in the normal gastric epithelial cell line GES-1, GAC cell lines (GT5, MKN45, N87, Snu-1, and Snu-16), and patient-derived PC cells (GA0518) by qPCR. The PVT1 level was higher in the GAC cell lines than in the GES-1 cell line; it was the highest in GA0518 PC cells (*p* < 0.001) and also upregulated in MKN45 cells (*p* < 0.001) (Figure 3A). We determined whether human PVT1-specific ASOs can suppress PVT1 expression in two cell lines GA0518 and MKN45 with PVT1 high expression. As shown in Figure 3B,C, two individual PVT1 ASOs (#5 and #6) significantly inhibited PVT1 expression in a dose-dependent manner in GA0518 cells; similarly, PVT1 ASO#5 and PVT1 ASO #6 dramatically suppressed PVT1 expression in MKN45 cells (Figure 3D,E).

### 2.4. Inhibition of PVT1 by PVT1 ASOs Suppressed Tumor Growth and Invasion In Vitro

**Figure 4:**
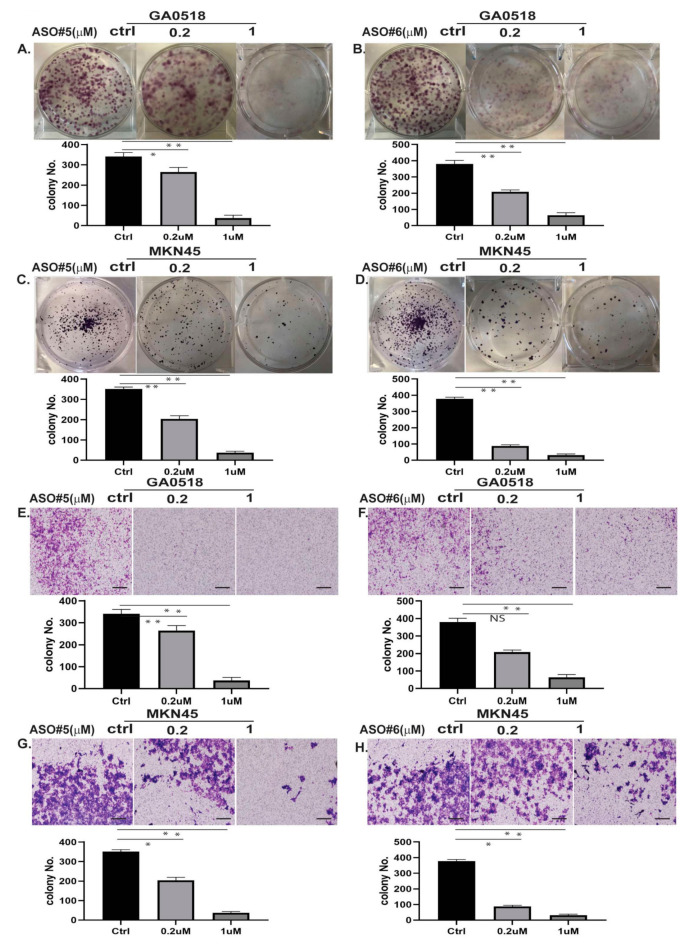
ASOs suppressed colony formation and cell invasion by inhibiting PVT1. (**A**,**B**) Colony formation of GA0518 cells was suppressed in a dose-dependent manner by ASO#5 (**A**) and ASO#6 (**B**). (**C**,**D**) Colony formation of MKN45 cells was suppressed in a dose-dependent manner by ASO#5 (**C**) and ASO#6 (**D**). (**E**,**F**) Cell invasion of GA0518 cells was suppressed in a dose-dependent manner by ASO#5 (**E**) and ASO#6 (**F**). (**G**,**H**) Cell invasion of MKN45 cells was suppressed in a dose-dependent manner by ASO#5 (**G**) and ASO#6 (**H**). scale bars = 40 μm. * *p* < 0.5; ** *p* < 0.01.

In vitro, colony formation and cell invasion assays were performed in MKN45 and GA0518 cells with high PVT1 levels were exposed to two ASOs at the dosage indicated to illustrate the functional effect of PVT1 inhibition by PVT1 ASOs in GAC cells. The results showed that two individual PVT1 ASOs significantly suppressed colony formation in a dose-dependent manner in GA0518 cells compared to in control ASO-treated cells (Figure 4A,B); similarly, both PVT1 ASO#5 and PVT1 ASO#6 decreased colony formation in a dose-dependent manner in MKN45 cells (Figure 4C,D). We further evaluated the effects of PVT1 ASOs on tumor cell invasion capacity using the transwell invasion assay and found that the number of invading cells was dramatically reduced when GA0518 cells were treated with PVT1 ASO#5 and PVT1 ASO#6 in a dose-dependent manner (Figure 4E,F). Similar results were found in MKN45 cells that had been treated with PVT1 ASO#5 and PVT1 ASO#6 (Figure 4G,H).

### 2.5. PVT1 ASOs Demonstrated Strong Antitumor Activity in an In Vivo PDX Model

**Figure 5:**
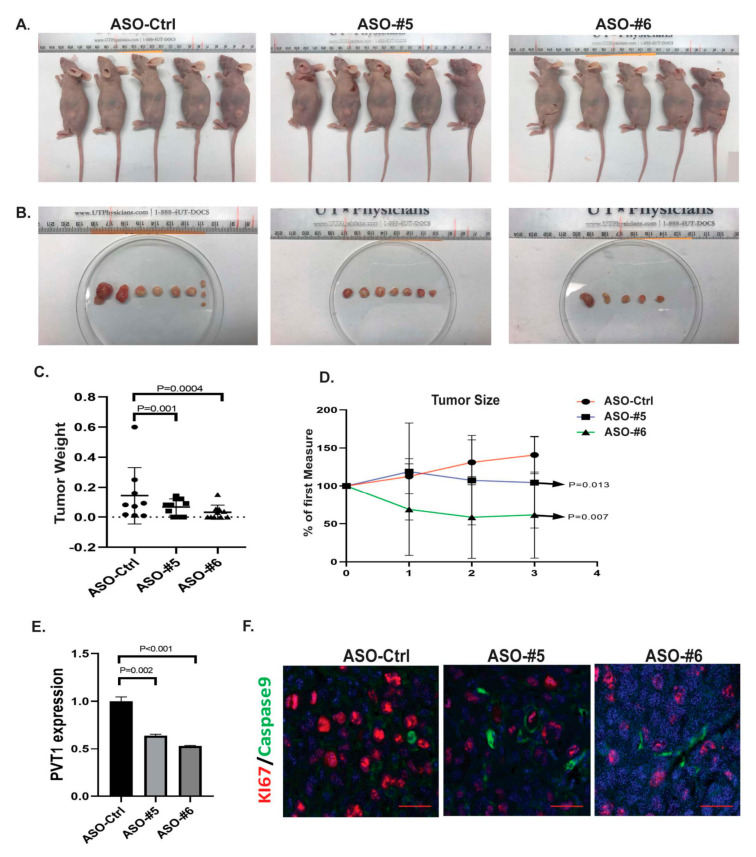
ASOs suppressed tumor growth in a GA0518 PDX model. (**A**,**B**) Mice were killed after the 3-week treatment. Tumor growth was observed and measured over 3 weeks of treatment. Representative mice and tumor images from ASOs-treated group and control group were demonstrated. (**C**,**D**) The tumor weights (**C**) and tumor volumes (**D**) of the ASO-treated group showed a significant reduction compared with those in the ASO control group. (**E**) PVT1 level was determined using q-PCR in mouse tumor tissues from PVT1 ASOs treated and control group. *P* value was shown in the Figure. (**F**) Immunofluorescent staining of proliferation marker KI67 and apoptotic marker Caspase 9 in PVT1 ASOs treated tumor tissues compared with ASO control tumor tissues were observed by confocal microscopy. Scale bars = 40 μm.

To further determine whether PVT1 inhibition by PVT1 ASO affects tumor growth in vivo, we injected GA0518 cells (with high PVT1 expression) subcutaneously into the back of the mice to form a PDX model. After 7 to 10 days, tumors formed in all mice injected, and were randomly assigned to one of the three groups: those treated with control ASO, PVT1 ASO#5, or PVT1 ASO#6 (50 mg/kg, 3 times/week, subcutaneously). Tumor growth and tumor volumes were observed and measured over a 3-week treatment period (Figure 5A–D). The volumes and weights of tumors from the PVT1 ASO-treated group were reduced by a significantly greater degree than were those of the control group (Figure 5C,D), while the mouse body weights among the groups were not significantly changed (not shown). We further validated the PVT1 level in treated mouse tissues and found expression of PVT1 was significantly reduced in tumors of the PVT1 ASOs treated group compared to the control group by q-PCR (Figure 5E). We also noticed that Ki67 cell proliferated cells were significantly reduced by PVT1 ASO#5 and PVT1 ASO#6, respectively, while cell apoptotic marker Caspase 9 was increased in PVT1 ASOs-treated group compared to ASO control group (Figure 5F). These results confirmed that PVT1 inhibition by PVT1 ASO efficiently suppressed tumor growth in vivo.

## 3. Discussion

Patients with advanced GAC are in desperate need of better therapeutic options. Novel targets and target strategies are needed. Mounting evidence has emerged that abnormal expression of lncRNA plays an important role in tumorigenesis, invasion, and metastasis. LncRNA PVT1 is a critical lncRNA that plays an oncogenic role in several tumor types [9,10,30,31], including GAC [15,19]. However, its precise role and its utility as a predictive marker and a therapeutic target in GAC remains unclear.

We have considerably extended our knowledge of the role of PVT1 in GAC in the current study. Using TCGA data, we first validated that genetic alterations (amplification and duplication) of PVT1 are common in GAC. We next evaluated PVT1 expression in normal tissue, primary GAC tissue, and PC tumor cells using qPCR and found that it was significantly upregulated in primary GAC tissue and PC cells compared to in normal tissue. PVT1 levels were further validated in a large cohort of TMA GAC patients using R-Scope ISH; PVT1 expression was significantly upregulated in GAC tissue compared to in adjacent normal tissues. High PVT1 expression was associated with larger tumor size, deeper tumor invasion, a higher frequency of lymph node metastases, and short survival duration. Most importantly, PVT1 ASOs efficiently inhibited the PVT1 level and suppressed GAC cell colony formation and invasion in vitro and delayed tumor growth in a PDX model in vivo.

The previous reports revealed that lncRNA PVT1 was upregulated in tumor tissues compared to adjacent normal tissues in GAC patients and was associated with lymph node metastases and a shorter survival [17,18,32,33,34]. Although these results are similar to ours, most of these studies used qPCR to detect PVT1 levels in normal and tumor tissues and the sample sizes were limited [18,33]. Thus, we have extended this previous work, we assessed PVT1 levels using R-Scope ISH in a large patient TMA (paired tumor and adjacent normal tissues from 792 patients). R-Scope technology represents cutting-edge ISH; it enables the simultaneous detection of the quantitative RNA signal and the morphological context of the tumor tissue architecture. Using R-Scope ISH, we directly visualized PVT1 expression; it was found mostly in the nucleus of tumor cells but was less enriched in other cell types or stromal cells. To our knowledge, this is the first study to use R-Scope ISH to detect the levels and localization of PVT1 in a large GAC TMA; our findings using R-Scope ISH demonstrated that high PVT1 expression in GAC tissues was associated with an aggressive phenotype, lymph node metastases, and shorter survival, consistent with the results of previous reports using qPCR [18,33]. Using R-Scope ISH in a large group of GAC patients, we validated that PVT1 is a poor prognostic marker for GAC.

We evaluated TCGA data and found that PVT1 upregulation was correlated with the CIN subtype of GAC, which has a poor prognosis and is often resistant to therapy [35]. Zhang et al. [36] found that PVT1 overexpression in GAC promoted the development of multidrug resistance, and Du et al. [37] reported that PVT1 was increased in 5-FU-resistant cells by upregulating BCL-2. In breast cancer, lncRNAPVT1 has been proven to play an oncogenic function by protecting cMYC protein, which plays emerging roles in cancer stemness and resistance to chemotherapy from phosphorylation-mediated degradation [38,39]. In the previous study, we also discovered a positive regulation and feedback between PVT1 and YAP1 in esophageal adenocarcinoma, which is involved in tumor development, growth, and homeostasis [10]. We assume that specific inhibition of PVT1 is able to increase sensitivity to chemotherapy drugs; thus, PVT1 ASOs may be successful in the clinic.

ASOs are an effective and feasible means of selectively targeting a gene of interest; four ASOs (fomivirsen, nusinersen, mipomersen, and eteplirsen) have already been approved by the FDA [40,41,42,43]. ASOs are known to cleave target RNA in both the cytoplasm and the nucleus, depending on RNase H1 [44]. Ionis Pharmaceuticals, a leader in ASO technology, has developed many ASOs, including PVT1 ASOs, for preclinical studies. Some of these ASOs have been used in clinical trials [45]. Through a collaboration between MD Anderson and Ionis, we determined whether PVT1 ASOs effectively target PVT1 in our system in vitro and in vivo. PVT1 ASOs effectively suppressed PVT1 levels in patient-derived cells and GAC cell lines, inhibited tumor cell colony formation and invasion in vitro, and suppressed tumor growth in vivo. These findings suggest that lncRNA PVT1 plays a role in oncogenesis and serves as a novel therapeutic target that can be effectively targeted by PVT1 ASOs in GAC patients.

## 4. Materials and Methods

### 4.1. Tissue Samples and Tumor Tissue Microarrays (TMAs)

A total of 17 pairs of GAC and normal tissues for quantitative real-time polymerase chain reaction (qPCR) were obtained from the First Affiliate Hospital of China Medical University (Shenyang, China), and malignant ascites cells from 14 peritoneal carcinomatosis (PC) patients were obtained from the Department of Gastrointestinal Medical Oncology at The University of Texas MD Anderson Cancer Center (MDACC; Houston, TX, USA). All research performed was approved by the Institutional Review Board of MDACC (code: LAB01-543). TMA containing paired GAC and normal tissues from 792 patients at the First Affiliate Hospital of China Medical University was studied locally. These patients had undergone total or subtotal gastrectomy and lymph node dissection between January 2009 and December 2014. None of the patients had undergone chemotherapy or radiotherapy prior to surgery. Patients provided written informed consent, and the study was approved by the ethics committee of China Medical University (code: (2015)119).

Patients’ detailed postoperative pathologic stage and demographic information were obtained from electronic medical records; it included age, sex, tumor location, tumor size, differentiation status, Lauren subtype, invasion depth, lymph node metastases, distant organ metastases, TNM stage, and vein invasion; these data are on 716 patients (319 PVT1 negative and 397 positive) and are shown in Table 1. We used the 7th American Joint Committee on Cancer staging manual TNM classification system for GAC. All patients were followed up via telephone inquiry or questionnaires. The follow-up period ranged from 2 to 98 months (median, 51 months).

### 4.2. Cell Lines and Reagents

The normal gastric epithelial cell line GES-1 and the GAC cell lines MKN45, GT5, N87, Snu-1, and Snu-16 were purchased from the American Type Culture Collection (ATCC, Manassas, VA, USA). GA051816 patient-derived cells were isolated from a patient-derived xenograft (PDX) that had been implanted with peritoneal carcinomatosis (PC) cells from a GAC patient. MKN45 and GT5 cells were grown in DMEM and the other cell lines including GA0518 were in RPMI-1640 medium with 10% fetal bovine serum at 37 °C in a 5% CO_2_ atmosphere. Cell lines were authenticated at the Characterized Cell Line Core Facility at MDACC every 6 months. ASOs for PVT1 were supplied by the Ionis Pharmaceuticals, Inc. (Carlsbad, CA, USA).

### 4.3. R-Scope ISH

For the FFPE TMA, tissue sections in 5-μm thickness were deparaffinized in xylene and dehydrated in an ethanol series. The sections were then incubated in citrate buffer (10 nmol/L, pH 6) at a boiling temperature (100 °C to 103 °C) for 15 min using a hot plate before being rinsed in deionized water and immediately treated with 10 μg/mL protease (Sigma-Aldrich, St. Louis, MO, USA) at 40 °C for 30 min in a HybEZ hybridization oven (Advanced Cell Diagnostics, Hayward, CA, USA).

Hybridization with target probes (RNAscope^®^ Probe- Hs-PVT1, 406951, ACD, CA, USA), a preamplifier, an amplifier, and a label probe and chromogenic detection were performed according to the protocol described previously [46]. FFPE samples were prepared and fixed according to American Society of Clinical Oncology/College of American Pathologists guidelines [14] (10% neutral buffered formalin for 6 to 72 h at ambient temperature), including pretreatment conditions such as citrate buffer temperature, pH, incubation time, and protease concentrations.

### 4.4. RNA Preparation and qPCR

Total RNA was extracted using TRIzol reagent (Invitrogen, Carlsbad, CA, USA). We performed reverse transcription using the SuperScript IV First-Standard Synthesis System (Invitrogen) and then performed qPCR using SYBR Select Master Mix (Applied Biosystems, Foster City, CA, USA) on the Applied Biosystems 7500 Fast platform (Applied Biosystems). We used the following PVT1 primers: F, 5′-TGAGAACTGTCCTTACGTGACC-3′; R, 5′-AGAGCACCAAGACTGGCTCT-3′. The internal control was GAPDH: F, 5′-TCTAGACGGCAGGTCAGGTC-3′; R, 5′-ACCCAGAAGACTGTGGATGG-3′. ΔΔCt values were used to determine relative expression levels as fold changes.

### 4.5. Invasion Assay

Transwell invasion assays were performed using 8.0-μm pore inserts (BD Biosciences, San Jose, CA, USA). A total of 2.5 × 10^4^ cells was suspended in 500 μL of serum-free medium and loaded into upper wells; the lower chambers were filled with 750 μL of complete medium with 10% FBS. Migration chambers were incubated in a 5% CO_2_ incubator at 37 °C for 36–48 h. Cells were then stained and counted in five random microscope fields.

### 4.6. Colony Formation

MKN45 and GA051816 cells were harvested by trypsin and seeded in the six-well plates (1000 cells/well). Cells were exposed to PVT1 ASOs with no transfection agent (free uptake) at the dosage indicated and cultured for 10–14 days to allow for colony formation. The culture medium was refreshed every 3 days. Once colonies were visible, cells were rinsed twice with phosphate-buffered saline, followed by fixation with 10% paraformaldehyde and staining with 3% crystal violet for 5 min. The number of colonies was photographed and counted using ImageJ software.

### 4.7. Patient-Derived Xenograft Model (PDX)

The PDX in vivo experiments were conducted in accordance with the guidelines of the MD Anderson Institutional Animal Care and Use Committee. In detail, 1 × 10^6^ patient derived GA051816 cells were subcutaneously injected into the back of nude mice (*n* = 5/group). After about 10 days, tumors were formed in the back of all the mice, then the mice were randomly divided into three groups (control ASO, ASO#5, and ASO#6), and then mice were subcutaneously injected with control ASO or PVT1 ASO (50 mg/kg) three times a week for at least 3 weeks. Tumor size was measured with a digital caliper (VWR International) once tumors had reached a visible size, and tumor volume was determined by the formula: tumor volume (mm^3^)  = [length (mm) × width (mm)^2^] × 0.52 [24].

### 4.8. Indirect Immunofluorescence

PDX tumors were fixed by formalin then embedded by paraffin. Indirect immunofluorescence staining with anti-Ki-67 (1:300) and anti-caspase-9 (1:100), then labeling with Alexa Fluor 555 (for Ki-67) and Alex Fluor -488 (for caspase-9) was performed as described [47,48]. Fluorescence was observed on a confocal microscope (FluoView FV500; Olympus America, Melville, NY, USA) and analyzed by CellQuest Pro software (BD Biosciences, Franklin Lakes, NJ, USA).

### 4.9. Statistical Analysis

Data were analyzed using Student’s *t*- and Fisher exact tests (for colony formation and cell migration assay). The Kaplan–Meier method was used to estimate the probability of survival. The log-rank test and Cox regression analyses were used to determine the association between markers and survival outcomes. Other assays are presented as mean ± SEM and represent the results of at least three experiments. Differences between groups were compared using a two-tailed Student *t*-test. Results were considered statistically significant if the *p* value was <0.05. All tests were performed using GraphPad Prism 8 software (GraphPad Software, Inc., San Diego, CA, USA) and SPSS.22 software (IBM, SPSS Software, Inc., Armonk, NY, USA).

## 5. Conclusions

In summary, for the first time, using R-Scope ISH in a large TMA, we validated that PVT1 upregulation in GAC (compared to in normal tissues) was associated with lymph node metastases and poor prognosis. Most importantly, we found that PVT1 ASOs efficiently suppressed PVT1 levels and provided obvious antitumor activity in in vitro and in vivo PDX models. PVT1 lncRNA seems to play a critical role in GAC progression and metastases that can be curtailed by specific ASOs.

## Figures and Tables

**Table 1:** Correlation between positive PVT1 expression and clinic-pathologic characteristics.

Characteristic	Total	No. of Patients		*p* Value
		−(319)	+(397)	
Age, years				0.362
Median	61			
Minimum-maximum	27–85			
Sex				0.204
Male	524	241	283	
Female	192	78	114	
Tumor size				0.017 *
Median (cm)	5			
Minimum-maximum (cm)	0.3–17			
Differentiation				0.785
Well	55	23	32	
Moderate	150	67	83	
Poor	511	229	282	
Lauren subtype				1.000
Diffused	519	231	288	
Intestinal	197	88	109	
T category				<0.001 **
T1	20	16	4	
T2	47	29	18	
T3	135	64	71	
T4	513	210	303	
N category				0.008 **
N0	163	73	90	
N1	152	77	75	
N2	151	84	67	
N3	245	82	163	
M category (M1 vs. M0)				0.496
M0	493	222	271	
M1	196	82	114	
TNM stage				0.124
I	31	21	10	
II	145	65	80	
III	344	151	193	
IV	196	82	114	
Vein invasion				0.185
+	6	3	3	
−	710	316	394	

* indicates *p* value < 0.5, ** indicates *p* value < 0.01; PVT1 upregulation is correlated with larger tumor size, invasion depth and lymph node metastasis.

**Table 2:** Univariate and multivariate Cox regression analyses.

PVT1	Univariate			Multivariate		
	HR	95% CI	*p* Value	HR	95% CI	*p* Value
Age (continuous)	1.014	1.002–1.027	0.024 *	1.015	1.002–1.029	0.028 *
Sex (male vs. female)	0.911	0.695–1.194	0.498			
Size (continuous)	1.099	1.048–1.151	<0.001 **	1.048	0.989–1.111	1.048
Differentiation			0.353			
Well	1		0.387			
Moderate	1.339	0.772–2.323	0.299			
Poor	1.423	0.855–2.368	0.175			
Lauren subtype (intestinal vs. diffused)	0.893	0.686–1.163	0.401			
Borrmann			0.008 **			
Early	1		0.030 *	1		0.218
1	1.362	0.538–3.453	0.515	1.774	0.190–16.580	0.615
2	0.415	0.150–1.145	0.089	0.432	0.043–4.364	0.477
3	1.175	0.580–2.379	0.654	1.281	0.151–10.879	0.821
4	1.590	0.729–3.469	0.244	1.421	0.161–12.558	0.752
T category			0.011 *			0.744
T1	1		0.021 *	1		
T2	0.656	0.254–1.692	0.383	0.331	0.036–3.039	0.328
T3	0.987	0.444–2.193	0.973	0.435	0.049–3.878	0.456
T4	1.391	0.655–2.953	0.390	0.437	0.050–3.847	0.456
N category			<0.001 **			<0.001 **
N0	1		<0.001 **	1		
N1	1.279	0.836–1.955	0.257	1.348	0.858–2.118	0.195
N2	1.448	0.956–2.194	0.081	1.256	0.783–2.015	0.344
N3	3.040	2.128–4.343	<0.001 **	2.751	1.847–4.098	<0.001 **
M category (M1 vs. M0)	2.073	1.624–2.646	<0.001 **	1.692	1.298–2.205	<0.001 **
Vein invasion (− vs. +)	0.969	0.241–3.894	0.964			
PVT1 (+ vs. −)	1.312	1.022–1.685	0.033 **	1.160	0.888–1.514	0.277

* indicates *p* value < 0.5, ** indicates *p* value < 0.01; Univariate Cox regression analysis showed upregulated PVT1 is correlated with worse outcome after operation, but the result of multi-variate Cox regression analysis showed PVT1 is not an independent prognostic factor.
